# Cancer Characteristics and Current Treatments of Patients with Renal Cell Carcinoma in Sweden

**DOI:** 10.1155/2015/456040

**Published:** 2015-10-11

**Authors:** Andreas Thorstenson, Ulrika Harmenberg, Per Lindblad, Benny Holmström, Sven Lundstam, Börje Ljungberg

**Affiliations:** ^1^Department of Surgical and Perioperative Sciences, Urology and Andrology, Umeå University, 901 87 Umeå, Sweden; ^2^Department of Molecular Medicine and Surgery, Section of Urology, Karolinska Institute, 171 76 Stockholm, Sweden; ^3^Surgical Intervention Trials Unit, Nuffield Department of Surgical Sciences, University of Oxford, Oxford OX3 7DQ, UK; ^4^Department of Oncology, Karolinska University Hospital, Solna, 171 76 Stockholm, Sweden; ^5^Department of Urology, Faculty of Medicine and Health, Örebro University, 701 82 Örebro, Sweden; ^6^Department of Urology, Akademiska University Hospital, 751 85 Uppsala, Sweden; ^7^Department of Urology, Sahlgrenska University Hospital, 431 45 Göteborg, Sweden

## Abstract

*Methodology*. Since the start in 2005 virtually all patients with newly diagnosed renal cell carcinoma (RCC) in Sweden are reported to the National Swedish Kidney Cancer Register (NSKCR). The register contains information on histopathology, nuclear grade, clinical stage, preoperative work-up, treatment, recurrence, and survival. *Results*. A total of 8556 patients with newly diagnosed RCC were registered in the NSKCR from 2005 to 2013 resulting in a coverage of 99% as compared to the Swedish Cancer Registry. The mean tumor size at detection decreased from 70 mm in 2005 to 64 mm in 2010. The proportion of patients who were incidentally detected increased. The proportion of patients with tumor stage T1a who underwent partial nephrectomy increased from 22% in 2005 to 56% in 2012. Similarly, the proportion of laparoscopically performed radical nephrectomies increased from 6% in 2005 to 17% in 2010. During the five years of follow-up 20% of the patients had a recurrence. *Conclusion*. Over the last decade there has been a trend of earlier detection and less advanced tumors at detection in patients with RCC. An increasing proportion of the patients undergo laparoscopic and nephron-sparing procedures.

## 1. Introduction and Objectives

In 2005 all the health care regions in Sweden joined the National Swedish Kidney Cancer Register (NSKCR). The intention of the NSKCR is to get reliable data regarding the health care offered to patients with renal cell carcinoma (RCC) and to support research on the subject. Other important aims for the NSKCR include the evaluation of the adherence to the national guidelines for RCC [1] and benchmarking and standardizing the work-up and management of patients with RCC. This register can now provide us with valid, population based data on virtually all patients with newly detected RCC in Sweden from the last 10 years. The current report summarizes obtained results during ten years with the NSKCR.

## 2. Material and Methods

### 2.1. The National Swedish Kidney Cancer Register

Sweden is divided into six health care regions, each consisting of two to seven counties, in total 21. Since January 2005, all the health care regions participate in the registration of patients in the NSKCR. All health care regions have their own local cancer register located in their Regional Cancer Center. The Regional Cancer Center of Stockholm-Gotland is responsible for the coordination of the regional registers to the NSKCR. The NSKCR is governed by a steering committee comprised of a urologist and a medical oncologist from each health care region as well as a national representative for histopathology.

All patients in Sweden with newly diagnosed RCC are reported to the respective health care region's cancer register. It is compulsory for health care providers to report all newly detected cancer patients to the register. The regional RCC-registries are regularly cross-linked with the regional cancer registries and missing patients can be identified and searched for. The identification of patients is efficient because every Swedish citizen has a personal identity number (PIN) [2]. According to the Swedish Data Inspection Board, PINs in national databases can be used for follow-up of the quality of health care. Ethical approval is thus not necessary for this purpose, provided that appropriate security measures are adhered to and the confidentiality rules of the Swedish law are applied [3]. Furthermore, there is a leaflet for the patients about the registration procedure which includes a possibility to opt out due to personal preference. Data from the regional registers are validated and checked by the respective Regional Cancer Center and reported once a year to the NSKCR. Updates and corrections of previous years are also included in the yearly reports. The coverage of the NSKCR is cross-checked with the Swedish Cancer Registry to which reporting of all new cases of cancer is mandated by Swedish law [4].

The patients of the NSKCR constitute a compiled clinical material of virtually all patients with newly diagnosed RCC in Sweden and are more thoroughly described in earlier publications [5, 6]. The register contains information on tumor characteristics, histological RCC subtype, nuclear grade, tumour size, and the TNM-classification according to the Union for International Cancer Control 2009 [7]. Further, information on preoperative work-up and surgical treatment is available in the register. Patients with localized disease at detection (M0) are followed-up after five years regarding disease recurrence and treatment. Histopathologic classification of RCC subtype is performed according to the WHO classification and for tumor grade the Fuhrman nuclear grading is used [8, 9]. In patients with more than one tumor, the largest tumor defines the tumor stage. Tumor size is measured by computed tomography (CT) or magnetic resonance imaging (MRI) and in rare cases from histopathological reports. Lymph node stage is based on CT and/or MRI examinations of the abdomen or histopathological findings after surgery. In order to be classified as N0, a negative CT of the abdomen and/or the finding of no pathologically enlarged lymph nodes at surgery are required. A preoperative chest CT is recommended in all patients to rule out pulmonary metastases. Until recently, the NSKCR did not contain information on patient characteristics such as ASA, performance status, and intraoperative and postoperative complications about neither treatments nor outcomes of patients with metastatic disease; however, such modules will be added.

### 2.2. Statistical Analysis

Descriptive statistics are provided in tables. Statistical tests were calculated using two-tailed *t*-tests and Chi-square tests. A two-tailed *p* value < 0.05 was considered statistically significant. The survival analyses using the log rank test were performed as relative survival. Relative survival is the observed survival in the patient group in relation to all comparable individuals from the total national population. Difference in relative survival is an approximation of the effect of the studied disease that represents the cause specific survival. Statistical analyses were performed with STATA, StataCorp., 2007, StataStatistical Software: Release 12.1. College Station, TX: StataCorp LP.

### 2.3. Validation and Quality Indicators

A validation of the register was made in 2009. Ten per cent of the registered patients from 2009 were sampled and data from the medical records was compared with the data that had been reported to the NSKCR. A mismatch regarding tumor size and grade was found in some of the patients. In order to correct this problem, alterations in the layout of the reporting module was made. It is no longer possible to enter an erroneous stage due to control of the input of data. If an erroneous stage is reported or if tumor size is reported as <1 cm or >15 cm, a validation question will occur during data entry. We plan to do repeated validations.

In order to increase the quality of care for patients with RCC and to allow for benchmarking, a number of quality indicators were measured in the NSKCR. Target levels of these quality indicators were set yearly and changed as appropriate. Some examples of quality indicators are the coverage of the register, the proportion of patients undergoing chest CT, partial nephrectomy, laparoscopic surgery, and centralization to high volume hospitals.

## 3. Results

### 3.1. Patients and Cancer Characteristics

A total of 8556 patients with newly diagnosed RCC were registered in the NSKCR between 2005 and 2013. This represents a coverage of 99% as compared to the Swedish Cancer Registry. Patients and cancer characteristics of the patients registered in the NSKCR are shown in Table 1 [10]. A proportion of 61% of the patients were male and 39% were female. The median age at diagnosis was 67 years (range 23–105 years) with no change in median (66-67 years) or mean (67-68 years) age through the 10 years. The most common RCC type was clear cell carcinoma which was diagnosed in 78% of the patients followed by papillary (12%), chromophobe (5%), and collecting duct (0.4%, Table 1) carcinoma.

**Figure 1:**
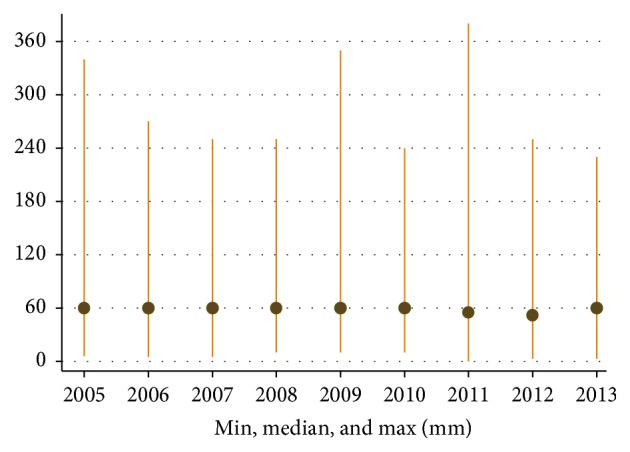
Variation of tumor size (mean and range) at the time of diagnosis (mm) in 2005–2013.

During the period we found a significant trend towards more incidental detection. In 2005, 43%, versus 48% in 2008 and in 2013 55%, of the patients were incidentally detected (*p* < 0.0001). More than half of the patients (52%) had stage T1 tumors at detection. There was a trend during the work with the NSKCR of an increase in the proportion of patients with stage T1a tumors and a decrease in patients with stage T1b tumors. During the study period the mean tumor size at detection gradually decreased from 70 mm in 2005 to 64 mm in 2010. Similarly, the median tumor size at detection decreased from 60 mm in 2005 to 55 mm in 2013 (Figure 1).

The recommended work-up for all patients with newly detected RCC included a chest CT for the evaluation of pulmonary metastases. The proportion of patients who underwent chest CT increased from 59% in 2005 to 90% in 2013 (*p* < 0.0001).

### 3.2. Metastatic Disease and Recurrent Disease

**Figure 2:**
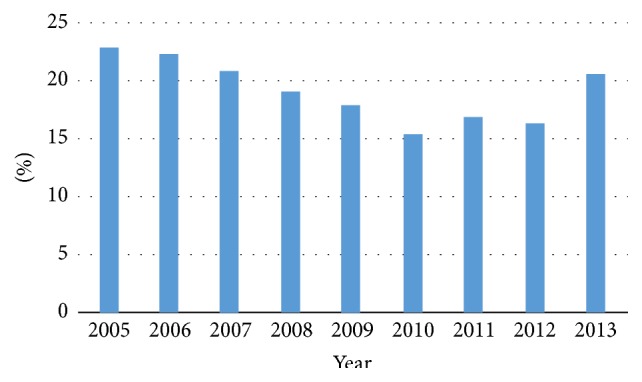
Percentage of patients diagnosed with metastatic renal cell carcinoma at primary diagnosis in relation to the total number of patients by year of diagnosis.

Metastatic disease (mRCC) at presentation was found in 5%, 9%, 21%, 37%, and 60% in patients with RCC stages T1a, T1b, T2, T3, and T4, respectively [6]. We have shown that even tumours as small as 1-2 cm have a malignant potential and may give rise to lymph node or distant metastatic spread [5]. The proportion of patients with mRCC at the time of diagnosis gradually decreased from 23% in 2005 to 16% in 2012. However, in 2013 the proportion of patients with mRCC increased to 19% (Figure 2).

The patients who were diagnosed with nonmetastatic RCC during 2005–2007 and treated with curative intention were followed up after five years. During the five years of follow-up, 20% of the patients suffered a recurrence. The most common location of recurrence was lung, followed by bone, lymph nodes, and liver.

### 3.3. Treatment

A total of 76% patients with RCC were treated with a curative intention. In the patients diagnosed in 2005–2011 radical nephrectomy (RN) was the most common treatment (74%) followed by partial nephrectomy (PN, 23%) and minimally invasive treatments such as cryotherapy and radiofrequency ablation were performed in 2% of the cases [11]. The most common minimally invasive treatment was radiofrequency ablation, which was performed in 80% of the cases. The proportion of PN in patients with RCC T1aN0M0 increased during the years of the register (*p* < 0.0001). In patients with RCC T1aN0M0, 22% underwent PN in 2005 and 56% in 2012.

An increased use of laparoscopic technique was noted during the years of work with the NSKCR. The laparoscopic approach was increasingly used both for RNs and PNs. The proportion of RNs laparoscopically performed increased from 6% in 2005 to 17% in 2010. During 2009–2013, 14% of the PNs were performed by laparoscopic technique and robotic assisted surgery was used in nearly one-third of these cases.

**Figure 3:**
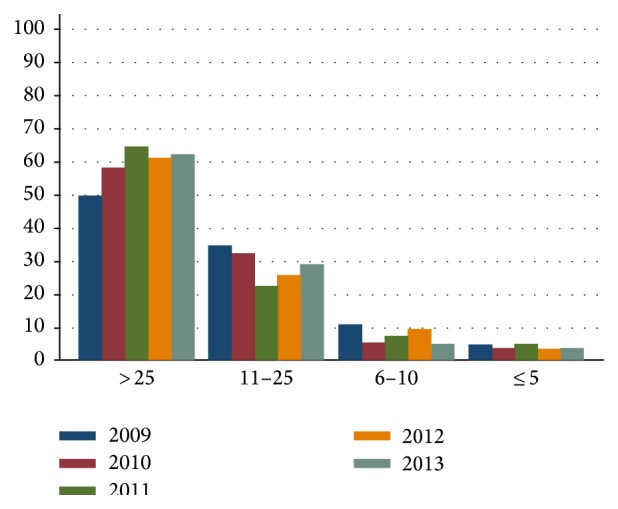
Number of nephrectomies in large volume hospitals (>25 nephrectomies per year) as compared to medium (6–25 nephrectomies per year) and small volume hospitals (≤5 nephrectomies per year).

During the last decade there has been a clear trend of an increasing proportion of patients with RCC that have been treated at large volume hospitals (>25 nephrectomies per year, Figure 3). In 2005, 34% of the patients underwent surgery at a large volume hospital and in 2009 it had increased to 56%. During 2009–2013, 100 patients per year in Sweden underwent surgery in a hospital performing <10 nephrectomies per year.

### 3.4. Survival

The survival at five years for the whole cohort of patients with RCC was 70%. We found no difference in relative survival between men and women. The relative survival was strongly connected to T and M stage. The relative survival at five years for patients with RCC M0 was 85% and for patients with RCC M1 20% [6].

## 4. Discussion

### 4.1. Accustomedness from NSKCR

The NSKCR has been in operation for a decade and virtually all patients with newly detected RCC in Sweden are registered. The register is continuously expanding and data is maturing. Several manuscripts emanating from the NSKCR are published ([5, 6, 10, 11], Table 2). We have found a steady trend of early detection in patients with RCC. Thereby, the tumors are smaller and less advanced at the time of diagnosis. This trend is probably not caused by an alteration in the disease per se but is more likely an effect of an expanding use of CT and other imaging techniques for various conditions. It has led to an increase of incidentally detected patients with RCC. The facts that incidentally detected patients have lower TNM-stage and smaller tumours compared to patients with symptomatic RCC have been shown in data from the NSKCR [7]. In a previous work by our group we demonstrated migration towards smaller tumours and lower stages and grades at detection for the period 1979–2001 [12].

The proportion of patients undergoing preoperative chest CT is continuously increasing during this last decade in Sweden. The registration itself, including the yearly reports from the NSKCR, probably stimulates stronger adherence to the national guidelines of RCC [1, 10].

In order to minimize errors due to misclassification one important aim for the NSKCR is to maintain histopathology reports that follow the Swedish recommendations [13]. In 2013, virtually all patients had a histopathology report that specified the RCC type, which contributes to the validity of the register. Furthermore, the distribution of the RCC types was similar between the health care regions which indicate good data quality.

PNs and laparoscopically performed RNs have become more widely used over the last decade. More than half of the patients with tumors ≤4 cm underwent PNs in 2012. This is the recommended treatment for T1a tumors in both the national and the EAU guidelines [1, 14]. Even though PN is underutilized in Sweden [11], we have found that nephron-sparing procedures are used more frequently year by year. The indications for ablative nephron-sparing procedures, such as cryotherapy and radiofrequency ablation, are less well established, recommended only in highly selected cases and their use differs considerably between the health care regions due to availability. In the Swedish guidelines for RCC we have chosen to recommend an increased use of laparoscopic nephrectomy without a fixed target level since they may be competitive with open PNs. Today, both PNs and RNs are increasingly performed by robot assisted laparoscopy, a trend that is likely to continue.

### 4.2. Strengths and Limitations

The strength of this material is that it includes virtually all patients diagnosed with RCC in Sweden. The data is truly population based and the risk for selection bias can be ignored. The data registration is performed prospectively and the data are continuously validated at the registration centers and thereby the likelihood of erroneous data is reduced.

Until now, there has been no specific information on patients with mRCC receiving systemic therapy. However, from now on there is an oncologic module which records oncological treatments. We also intend to include patient reported outcome measures (PROM) as well as a detailed registration of surgical variables and surgical complications occurring within 90 days.

## 5. Conclusions

In conclusion, we find a continuous trend of early detection and migration towards less advanced tumors at presentation in patients diagnosed with of RCC in Sweden during the last decade. An increasing proportion of the patients undergo laparoscopic- and nephron-sparing procedures.

## Figures and Tables

**Table 1:** Patient and cancer characteristics in patients with RCC registered in the NSKCR from 2005 to 2013.

		Number of patients (%)
Total number of patients		8556 (100%)

Gender	Male	5256 (61%)
	Female	3300 (39%)

Age at diagnosis	Median 67 yrs (Range 23–105 yrs)	

Stage	T0	3 (0%)
	T1a	2511 (29%)
	T1b	2007 (23%)
	T2	1506 (18%)
	T3	2017 (24%)
	T4	287 (3%)
	Tx	213 (2%)
	Missing data	12 (0%)

Fuhrman grade^*∗*^	G1	926 (12%)
	G2	3496 (44%)
	G3	2072 (26%)
	G4	705 (9%)
	GX	657 (8%)
	Missing data	178 (2%)

Histopathology^*∗*^	Clear cell	6298 (78%)
	Papillary	957 (12%)
	Chromophobe	399 (5%)
	Collecting duct	33 (0.4%)
	Not possible to classify	156 (2%)
	Other kidney cancers	147 (2%)
	Missing data	44 (1%)

^*∗*^Data on histopathology was not available in 522 patients.

**Table 2:** Publications based on data from the NSKCR.

Reference	Journal	Year	Topic	Main findings
Guðmundsson et al. [5]	European Urology	2011	Metastatic potential in small renal tumors	Even small tumors (1-2 cm) have a malignant potential

Thorstenson et al. [6]	Scandinavian Journal of Urology	2014	Tumor characteristics and surgical treatment	A significant decrease in tumor size and metastatic disease at presentation

Ljungberg et al. [11]	Scandinavian Journal of Urology	2014	Surgical treatment of T1c tumours	Partial nephrectomy was underutilized in 2005–2011 (23% underwent PN)

Thorstenson et al. [10]	Scandinavian Journal of Urology (in press)	2015	The impact of quality indicators on the compliance to guidelines for RCC	Quality indicators in the NSKCR increased the compliance to the national guidelines for RCC
